# The Anti-Inflammatory Potential of an Ethanolic Extract from *Sarcopoterium spinosum* Fruits for Protection and/or Counteraction against Oxidative Stress in Dysfunctional Endothelial Cells

**DOI:** 10.3390/ijms25031601

**Published:** 2024-01-27

**Authors:** Hawraa Zbeeb, Francesca Baldini, Lama Zeaiter, Laura Vergani

**Affiliations:** 1 Department of Earth, Environment and Life Sciences (DISTAV), University of Genova, Corso Europa 26, 16132 Genova, Italy; 2 Nanoscopy and NIC@IIT, Istituto Italiano di Tecnologia (IIT), 16152 Genova, Italy

**Keywords:** *Sarcopoterium spinosum* fruit extract, polyphenols, antioxidant activity, anti-inflammatory activity, wound healing

## Abstract

Plants and plant extracts are a relevant source of bioactive compounds widely employed as functional foods. In the Mediterranean area, the shrub *Sarcopoterium spinosum* is traditionally used as an herbal medicine for weight loss and a diabetes treatment. Inflammation is a protective mechanism involved in the development of many pathological conditions, including cardiovascular diseases. The present study aimed to investigate in vitro the antioxidant and cytoprotective properties of an ethanolic extract from *S. spinosum* fruits (SEE) in a cellular model of endothelium dysfunction. Corilagin and quercetin are two polyphenols abundant in SEE and were tested for comparison. The exposure of HECV cells for 24 h to 30 µM hydrogen peroxide (H_2_O_2_) lead to an oxidative stress condition. When HECV cells were treated with 10 µg/mL of SEE or single compounds after or before the oxidative insult, the results showed their ability to (i) decrease the reactive oxygen species (ROS) production quantified using fluorometric analysis and the lipid peroxidation measured with a spectrophotometric assay; (ii) rescue both the glutathione reduced to oxidized (GSH/GSSG) ratio and nitric oxide impair and the protein denaturation; and (iii) accelerate the wound repair measured using a T-scratch assay. Taken together, our findings indicate that the ethanolic extract from *S. spinosum* fruits could be a potential candidate for nutraceutical application.

## 1. Introduction

Inflammation is a protective mechanism of the organism in response to external and internal stimuli caused by mechanical, chemical, or biological stresses [1]. Oxidative stress plays a crucial role in the development and perpetuation of inflammation and contributes to the pathophysiology of a number of diseases, such as cardiovascular diseases, diabetes, metabolic syndrome, degenerative processes, and cancer [2]. Oxidative stress refers to the unbalance between the generation of pro-oxidant species and antioxidant defenses, which may occur for both increasing the production of oxidant molecules and/or the impairment of the antioxidant system [3]. A physiological production of reactive oxygen species (ROS) and free radicals results from many cellular processes, such as mitochondrial respiration and metabolism, however, when the production overpasses the antioxidant defense, this may lead to oxidative stress and tissue damage [4]. The denaturation of tissue proteins is well documented in inflammatory diseases and it is considered a marker for inflammation [5].

The vascular endothelium is the largest organ in the body and plays anti-inflammatory and anticoagulant roles, maintains vascular tones, and acts as a physiological barrier to prevent blood cell adhesion [6]. Endothelial dysfunction is characterized by decreased production and/or local bioavailability of nitric oxide (NO) and an excess production of ROS [7]; in turn, endothelium dysfunction may trigger a lot of pathological pathways such as cardiovascular diseases (CVD), typically associated with diabetes and obesity [8,9,10].

To control inflammation, both steroidal and non-steroidal anti-inflammatory drugs (NSAID) can be used, but they may result in undesirable side effects. This has led to increasing interest in the research of bioactive compounds with anti-inflammatory activity extracted from natural sources, as they can offer certain advantages compared with synthetic drugs, such as the low incidence of adverse effects in the patient.

Polyphenols (PPs) are phenyl propanoids synthetized by plants as secondary metabolites, and are found largely in the fruits, vegetables, and cereals [11]. More than 8000 phenolic molecules have been identified, and polyphenols must contain at least one aromatic nucleus and one or more -OH groups. The Mediterranean diet is characterized by a high consumption of foods rich in polyphenols, and it is associated with health promotion [12]. In particular, polyphenols are potent antioxidant, anti-inflammatory, antimicrobial, and cardio-protective agents [13]. Towards the end of the 20th century, epidemiological studies and associated meta-analyses strongly suggested that the long-term consumption of diets rich in plant polyphenols protects against metabolic disorders and CVD [14,15].

*Sarcopoterium spinosum*, Bilan in Arabic, is a chamaephyte of the *Rosaceae* family, which is largely employed as a medicinal plant in the Mediterranean region. Ethnopharmacological studies reported the traditional usage of *S. spinosum* extracts for the treatment of several disorders, especially diabetes [16,17], pain relief [18], asthma, kidney stones, and poisoning [19]. The majority of surveys focused on the aqueous extract from *S. spinosum* roots [20,21], whereas the biological effects of the fruits were less investigated. In a recent study of our group, different extracts from *S. spinosum* fruits were compared in terms of their phytochemical activity and lipid-lowering effect in steatotic hepatocytes [22]. As a last consideration, the folk habits in using medicinal plants, *S. spinosum* in particular, lead the Lebanese population to consume them as infusions/tea as therapies for specific illnesses, or to drink them daily as a healthy habit for disease prevention. Therefore, in our study, we tried to mimic both these modalities of intake.

The primary objective of the present study was to investigate the therapeutic potential of *S. spinosum* fruits by testing the antioxidant, anti-inflammatory, and cytoprotective activity of the ethanolic extract (SEE) and its main polyphenols, corilagin (Cg) and quercetin (Qu). The cellular model of endothelial dysfunction consisted of human endothelial cells exposed to an oxidative insult such as H_2_O_2_. In an attempt to mimic the different modalities of intake, the experimental design included two different treatment protocols: In one, the cells were pre-treated with the SEE or PPs before the H_2_O_2_ insult; in the other one, the cells were, firstly, insulted with H_2_O_2_ and then treated with the SEE or PPs. A panel of oxidative stress markers was evaluated, including the cytosolic ROS production, the NO release, the lipid peroxidation levels, and the glutatione reduced to oxidized_(GSH/GSSG) ratio. Moreover, the protective ability of the SEE was tested as a protector against protein denaturation and as a promoter of wound healing.

Therefore, this study marks the initial attempt to comprehensively explore the biological effects of *S. spinosum* fruits on a cell model of endothelial dysfunction by employing a multifaceted approach. Of note, we focused on *S. spinosum* fruits rather than on roots in order to uphold the plant’s integrity and contribute to eco-sustainability. Our findings suggest that the ethanol extract, featuring key polyphenols such as corilagin and quercetin, is endowed with significant anti-inflammatory and antioxidant activities that ameliorated the dysfunction of endothelial cells. This positions the SEE as a promising nutraceutical candidate with potential applications in the prevention and defense against diseases triggered by oxidative stress.

## 2. Results

### 2.1. Cytoprotective Activity of SEE on Endothelial Cells

As outlined in our previous publication [22], the ethanolic extract from *S. spinosum* fruits boasts a rich profile of polyphenols, encompassing a total of 24 distinct compounds, with 17 of them successfully identified [22]. The ellagitannin family was found to be the most abundant group of PPs in the extract (approximately 50.8%). Triterpenes and flavonoids were also present in significant amounts (about 11.9% and 7% of the extract, respectively). In an attempt to improve phenolome identification, we carried out a quantitative analysis using ImageJ 2.9.0/1.54c software [23] to calculate the relative area of each peak, allowing us to obtain a percentage composition of the different compounds within the SEE (Table 1).

**Figure 1:**
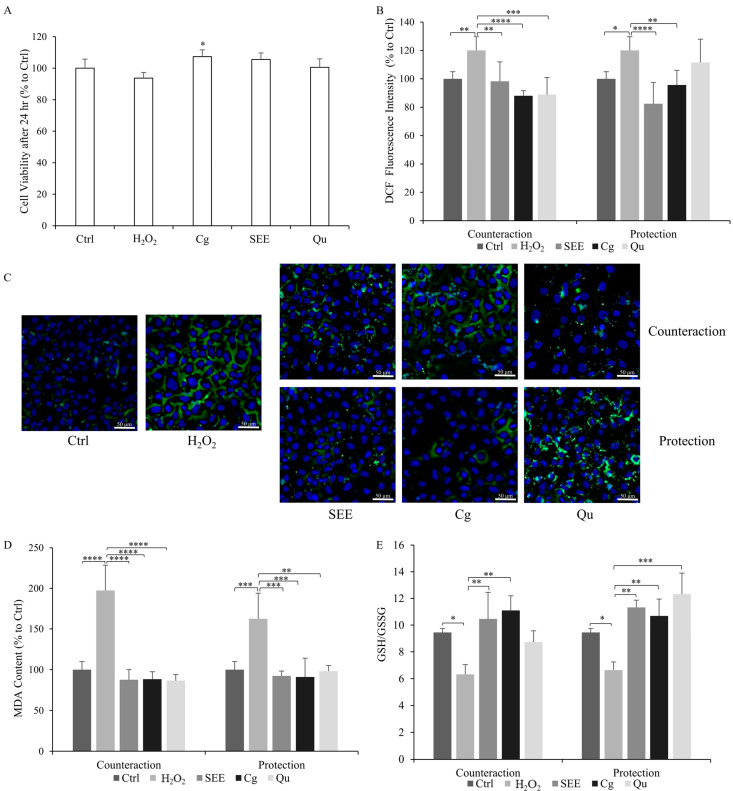
Antioxidant activity of the *S. spinosum* fruit ethanolic extract. Effects of 24 h treatment with either 10 µg/mL of extract (SEE), corilagin (Cg), or quercetin (Qu) on H_2_O_2_-insulted HECV cells evaluated in terms of (**A**) cell viability evaluated using MTT assay; (**B**) intracellular ROS level quantified using spectrofluorometric assay on DCF-stained cells and expressed as percent relative to control after normalization for total proteins; (**C**) fluorescence microscopy of DCF/Hoechst stained cells; (**D**) intracellular level of MDA (pmol MDA/Ml × mg of sample protein) quantified using TBARS assay and expressed as percent relative to control after normalization for total proteins; (**E**) GSH/GSSG ratio quantified using colorimetric kit. Values are mean ± S.D. from at least three independent experiments. Statistical significance between groups was assessed using ANOVA followed by Tukey’s test. Symbols: * *p* ≤ 0.05, ** *p* ≤ 0.01, *** *p* ≤ 0.001, **** *p* ≤ 0.0001.

As a preliminary step, we assessed if SEE, single PPs, and H_2_O_2_ affect the viability and/or proliferation of HECV cells in order to identify the concentrations at which they could be employed. Any cytotoxicity on HECV cells after 24 h was excluded for both the oxidative insult (30 µM H_2_O_2_) as well as for the SEE and the single polyphenols corilagin (Cg) and quercetin (Qu) at the dose of 10 μg/mL (Figure 1A). Notably, the exposure of HECV cells to corilagin resulted in a slight stimulation of cell proliferation (+7%, *p* ≤ 0.05 compared to Ctrl).

### 2.2. Antioxidant Activity of SEE on Dysfunctional Endothelial Cells

As expected, the exposure of HECV cells to 30 µM H_2_O_2_ led to a significant increase in ROS production (+20% vs. untreated control; *p* ≤ 0.05) (Figure 1B). Both SEE and single PPs caused a significant counteraction effect when administered after the H_2_O_2_ insult, resulting in a notable reduction in ROS levels compared to the H_2_O_2_-insulted cells. Specifically, reductions of −22% for SEE (*p* ≤ 0.01), −33% for Cg (*p* ≤ 0.0001), and −22% for Qu (*p* ≤ 0.001) were observed. Furthermore, both SEE and single PPs were also effective in protecting cells against oxidative stress development when administered before the H_2_O_2_ insult, resulting in lower ROS production in cells pretreated with SEE (−24%, *p* ≤ 0.001) and Cg (−24%, *p* ≤ 0.01) compared to H_2_O_2_-insulted cells. Of note, Qu pretreatment did not yield a significant difference. Upon comparing the counteraction and prevention conditions, both SEE and Cg proved effective as antioxidant agents. Notably, Cg exhibited maximal activity in the counteraction condition. The changes in ROS production as a function of the treatments were visualized in parallel using fluorescence microscopy (Figure 1C).

As ROS over-production may trigger lipid peroxidation of the cell membrane, the byproduct malondialdehyde (MDA) was quantified using a TBARS assay (Figure 1D). The MDA level markedly increased in H_2_O_2_-insulted cells compared to the control (+63%, *p* ≤ 0.001 for the protection condition, and +97%, *p* ≤ 0.0001 for the counteraction condition). In the counteraction condition, we observed a significant antioxidant effect for all the compounds, which reduced the MDA level by −110% (SEE), −109% (Cg), and −111% (Qu) (*p* ≤ 0.0001) compared to the H_2_O_2_-insulted cells. In the protection condition, both SEE and single PPs were able to stabilize and prevent the increase in MDA levels. The results showed lower MDA levels when cells were pre-treated with SEE and PPs before the H_2_O_2_-insult: −70% (SEE), −72% (Cg), and −64% (Qu) (*p* ≤ 0.01) compared to the H_2_O_2_-insulted cells. Therefore, in both experimental conditions, the SEE elicited the antioxidant effects, but the counteraction condition was more effective in reducing lipid peroxidation.

In order to balance the redox status, the reduced glutathione (GSH) can be oxidized to glutathione disulfide (GSSG). Figure 1E shows the changes in the GSH/GSSG ratio as a function of the treatments. We observed a significant reduction (*p* ≤ 0.05) in the GSH/GSSG ratio in H_2_O_2_-insulted HECV cells (from 9.5  ±  0.3 in control cells to 6.3 ± 0.7 in H_2_O_2_-insulted cells in the counteraction condition, and 6.6 ± 0.6 in the protection condition). The GSH/GSSG ratio was significantly increased (*p* ≤ 0.01) by the post-treatments with SEE and Cg (10.5 ± 2 for SEE and 11.1 ± 1.1 for Cg). On the other hand, in protection conditions, all compounds prevented the H_2_O_2_-induced decrease in the GSH/GSSG ratio (11.3 ± 0.5 for SEE, 10.7 ± 1.3 for Cg, and 12.3 ± 1.6 for Qu) respect to H_2_O_2_-insulted cells (*p* ≤ 0.01 and *p* ≤ 0.001, respectively). Of note, all the compounds were more potent in the rescue of the GSH/GSSG ratio in the protection conditions.

### 2.3. Anti-Inflammatory Activity of SEE on Dysfunctional Endothelial Cells

The anti-inflammatory potential of SEE was evaluated in vitro using a cell-free assay measuring the inhibition of the thermal denaturation of albumin. Protein denaturation refers to a process in which the proteins lose their 3D structure, leading to impairment/loss of their biological functions.

**Figure 2:**
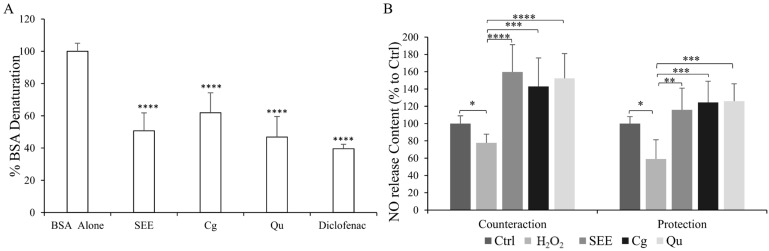
Anti-inflammatory activity of the *S. spinosum* fruit ethanolic extract. Influence of 10 µg/mL of either extract (SEE), corilagin (Cg), or quercetin (Qu) as an anti-inflammatory agent. We assessed (**A**) in vitro cell-free inhibition of heat-induced BSA denaturation at 75 °C using diclofenac sodium as a reference; (**B**) NO production in H_2_O_2_-stimulated HECV cells quantified in the medium as μmol NaNO_2_/mg sample protein with the Griess reaction. All values are expressed as % of control. Values are mean ± S.D. from at least three independent experiments. Statistical significance between groups was assessed using ANOVA followed by Tukey’s test. Symbols: * *p* ≤ 0.05, ** *p* ≤ 0.01, *** *p* ≤ 0.001, **** *p* ≤ 0.0001.

The possible inhibitory effect on protein denaturation was measured for all compounds at 10 µg/mL concentration. Diclofenac was employed as a positive control as it showed the highest ability to protect BSA from thermal denaturation (inhibition of 60%; *p* ≤ 0.0001). Among PPs, a similar strong effect was observed for Qu and SEE (inhibition of 53% and 49% vs. BSA alone, respectively; *p* ≤ 0.0001) while Cg showed a lower protective activity (inhibition of 38%; *p* ≤ 0.0001) (Figure 2A).

The anti-inflammatory potential was also evaluated by measuring the NO release in H_2_O_2_-insulted HECV cells (Figure 2B). Upon insult with H_2_O_2_, the HECV cells showed a decrease in NO release (−22% in the counteraction condition and −41% in the protection condition compared to control; *p* ≤ 0.05). Both the single PPs and the SEE rescued the impairment in the H_2_O_2_-induced NO release. In the counteraction condition, we observed a significant increase in the NO levels by +82%, (SEE), +65%, (Cg), and +74% (Qu) compared to only H_2_O_2_-insulted cells. Also, for the protection condition, the NO release increased significantly by +57%, (SEE), +65% (Cg), and +67%, (Qu) compared to the H_2_O_2_-insulted cells. (Figure 2B). Of note, in the counteraction condition, SEE was the most effective in restoring the NO release.

### 2.4. Promoting Effect of SEE on the Wound Repair of Endothelial Cells

**Figure 3:**
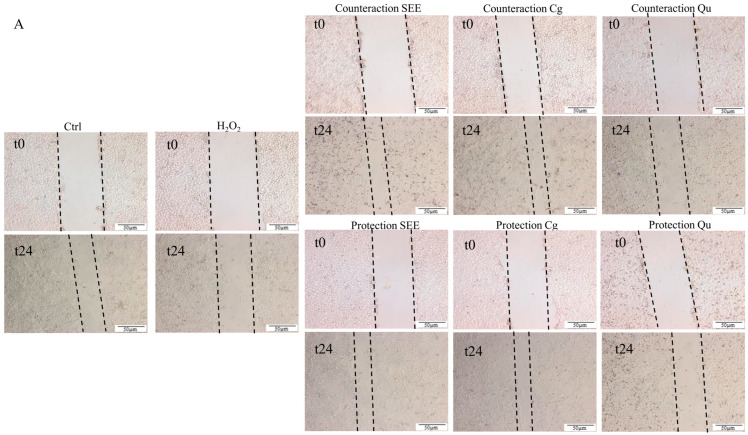

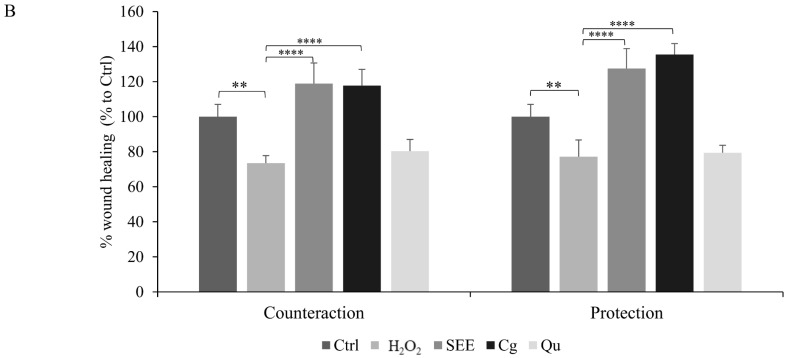
Wound repair-promoting effect of the *S. spinosum* fruit ethanolic extract. Influence of 10 µg/mL of either extract (SEE), corilagin (Cg), or quercetin (Qu) on cell migration and wound repair measured using the T-scratch assay on H_2_O_2_-stimulated HECV cells. (**A**) The wound width was marked by dotted lines in representative images acquired using an inverted Olympus IX53 microscope (Olympus, Milan, Italy) and captured with a CCD UC30 camera and digital image acquisition software (cellSens standard 1.9; (**B**) Histograms representing the percentage of wound healing 24 h after inducing the wound (% to Control). Values are mean ± S.D. from at least three independent experiments. Statistical significance between groups was assessed using ANOVA followed by Tukey’s test. Symbols: ** *p* ≤ 0.01, **** *p* ≤ 0.0001.

The T-scratch assay is typically utilized to quantify cellular migration on two-dimensional (2D) surfaces over time upon treatment. Figure 3 reports the images and the histograms for all the samples with the wound width at t24 expressed as a % with respect to the original wound width at t0 from the scratch. At t24, the control HECV cells showed a reduction in the wound width of −54%, while in the H_2_O_2_-insulted cells, the wound width was reduced only −25% (counteraction condition) and −36% (protection condition). This indicates a slowdown in the healing process due to the H_2_O_2_ insult around −25% (*p* ≤ 0.01) in both the counteraction and protection conditions compared to normal cells. The treatment with PPs resulted in an average acceleration of wound repair compared to positive and negative controls. Of note, a significant acceleration in the wound healing process was observed only when SEE and Cg were added before or after the H_2_O_2_ insult, while quercetin did not significantly affect wound repair. In the counteraction condition, both SEE and corilagin reduced the wound width to a similar extent (−60% and −62%, respectively). Similar results were observed in the protection condition, with SEE and Cg reducing the wound width to a similar extent (−64% and −68%, respectively). These data indicate that both SEE and Cg trigger a significant acceleration of wound repair in both the counteraction condition (+46% and +45%, respectively, *p* ≤ 0.0001) and in the protection condition (+50% and +59%; *p* ≤ 0.0001) compared to the H_2_O_2_-insulted cells.

## 3. Discussion

Reinforcing the defenses of the vascular endothelium against oxidative stress and inflammation has been proposed as a viable option for reducing the onset and progression of cardiovascular disease and other pathologies such as diabetes, metabolic syndrome, and degenerative processes. The main finding of the present study is the demonstration that the ethanolic extract from *S. spinosum* fruits, extremely rich in polyphenols, protects endothelial cells against the oxidative stress generated by an oxidant insult. The antioxidant activity is sustained by both the direct scavenging of the excess ROS and by the rescue of the GSH/GSSG and NO impairment. In addition, the cytoprotective action is accompanied by an anti-inflammatory activity, protecting against tissue protein denaturation, and a proliferative effect accelerating the wound healing process.

*S. spinosum* is a medicinal plant traditionally used as an anti-diabetic remedy in Lebanon [17]. Although the anti-diabetic activity of *S. spinosum* root extracts is reported in different studies [20,21], the beneficial effects of its aerial parts, especially its fruits, have been poorly elucidated in the past. In a recent paper published by our group [22], the ethanolic extract from *S. spinosum* fruits has been extensively investigated in terms of phenolome profile and biological activity using a model of steatotic hepatocytes. Indeed, the *S. spinosum* ethanolic extract is rich in ellagitannins (such as corilagin), flavonoids (such as quercetin), and triterpenoids [22]. Among three different extracts (water, ethanol, and boiling water) the ethanolic extract resulted in the most potent radical scavenger action using in vitro cell-free assays such as 2-2′-azino-bis(3-ethylbenzo-thiazoline-6-sulphonate) (ABTS) assay, and it was chosen for further investigations as a lipid-lowering and antioxidant agent against moderate hepatic steatosis. For this reason, we decided to expand the investigation by studying the possible ability of SEE in protecting the vascular endothelium. Therefore, the antioxidant and cytoprotective abilities of SEE were investigated using a cellular model of endothelium dysfunction.

Endothelium dysfunction can be defined as an alteration of the endothelium physiology towards a pro-inflammatory and pro-thrombotic state. This condition is linked to cardiovascular diseases, diabetes, autoimmune diseases, and bacterial and viral infections [8,10]. Endothelium dysfunction is characterized by inflammation, altered NO production and bioavailability, ROS over-production, and lipid peroxidation [24]; these markers were considered and investigated in our study.

To mimic in vitro the endothelium dysfunction occurring during CVD, HECV cells were exposed to H_2_O_2_, which triggers oxidative stress inside the cells [25,26]. To test the beneficial potential of the SEE, Cg and Qu we used in two experimental conditions. In the counteraction condition, we first triggered oxidative stress by exposing cells to H_2_O_2_, and then treated the cells with SEE or individual PPs. In the protection condition, we pre-treated the cells with SEE or individual PPs and then insulted them with H_2_O_2_. These conditions were designed to mimic the traditional modalities of *S. spinosum* intake by people who can use this plant to prevent and/or treat various disorders. Indeed, the Lebanese population may consume an infusion of *S. spinosum* parts as a therapy for pain or specific illnesses (such as diabetes), or may drink daily the *S. spinosum* infusion as a healthy habit for disease prevention.

In this study, we observed that, as a consequence of H_2_O_2_ insult, the endothelial cells developed oxidative stress, which was counteracted/prevented by the SEE, or the single compounds. In both experimental conditions, a significant decrease in intracellular ROS production was observed in situ with fluorescence microscopy and fluorimetric analysis. Also, the ROS-related lipid peroxidation was significantly decreased by SEE and single compounds, compared to the H_2_O_2_-treated cells. The efficacy of the SEE and its single compounds corilagin and quercetin probably depends on their direct action as scavengers of hydrogen peroxide due to the presence of phenolic groups that are known to be able to transform H_2_O_2_ into water by donating electrons [27]. Of note, both the SEE and the corilagin and quercetin were able to counteract ROS production and lipid peroxidation in steatotic hepatocytes [22].

Reduced glutathione (GSH) is the main non-enzymatic intracellular antioxidant and it is a marker for oxidative stress [28]. GSH acts by reducing peroxides (hydrogen and lipid peroxides) through its oxidation to GSSG by acting as a co-substrate of glutathione peroxidase [29]. In normal cells, the concentration of GSH typically ranges between 1 and 10 mM, and in different oxidative models, the GSH/GSSG ratio markedly decreases [30]. Our results showed a decrease in the physiological GSH/GSSG ratio in dysfunctional endothelial cells, and all the treatments, except quercetin, were able to rescue the impairment in the GSH/GSSG ratio in both experimental conditions (protection and counteraction).

Endothelial-derived NO is a versatile molecule with a large impact on many physiological functions. In tissue inflammation, an increased production of NO by macrophages has been reported [31], whereas in the vascular endothelium, a decreased production of NO occurs [1]. Our results show a decrease in NO release in H_2_O_2_-stimulated cells. The primary process that decreases the bioavailability of vascular NO is associated with the NO breakdown through oxidation by ROS, mainly superoxide [32]. Of note, NO regulates the permeability of the endothelial barriers, acting as an anti-inflammatory agent [33]. Our results proved the ability of the SEE, and the single phenolic compounds, to restore NO bioavailability in line with previous studies [34].

Metabolic disorders typically lead to increased protein denaturation in many tissues that results in the generation of autoantigens and the amplification of inflammation [35]. Therefore, agents that can prevent protein denaturation could be of great interest in anti-inflammatory strategies. Several non-steroidal, anti-inflammatory agents have been reported to preserve/safeguard albumin from thermal denaturation [5]. Our findings show that both the SEE and the single polyphenols were effective in protecting albumin from thermal denaturation, thus suggesting that the extract might be a stabilizing agent with anti-inflammatory potential.

Cell migration holds a significant role in a broad spectrum of physiological and pathological events [36], and it encompasses essential aspects such as development, angiogenesis, inflammatory responses, wound healing, and tumor invasion [37]. Wound healing is a complex event, where a variety of cell types interact, playing various functions. The endothelial cells have a special role, as they undergo a series of morphological and functional alterations during wound healing [38]. When the SEE was tested for its potential in wound repair, we observed that both the extract as well as corilagin significantly accelerated wound closure when compared to the positive and negative controls. As quercetin was not effective in accelerating wound repair, we can hypothesize that the extract might act mainly depending on its high content in corilagin. Indeed, we observed that corilagin increased cell proliferation in the MTT assay. Interestingly, our findings are in line with the results reported for different species of the Rosaceae family. In particular, both a methanolic extract from *Rubus imperialis* was able to promote wound healing in artificially wounded L929 fibroblasts [39], and a methanol bark extract from *Prunus africana* demonstrated wound-healing activity in a mouse model [40]. According to the general idea, the wound-healing effects of our extract and corilagin could be related to their ability to control oxidative stress [41].

Taken together, our findings highlight the promising efficacy of *S. spinosum* fruits in the form of an ethanol extract against endothelial dysfunction. Moreover, SEE was effective in two modalities of administration: It could counteract oxidative stress and inflammation by restoring the physiological parameters that had been impaired in dysfunctional cells, but it also actively prevented the onset of oxidative stress when it was used as a pre-treatment. These compelling outcomes strongly suggest that incorporating these compounds into a regular dietary regimen or utilizing them during periods of illness can indeed yield significant benefits and prove to be effective strategies for maintaining endothelial health.

The outcomes of this study could pave the way for future in vivo investigations into disease treatments utilizing natural antioxidants like SEE, Cg, and Qu as nutraceutical supplements. This further exploration aims to substantiate and expand upon the biological effects observed in cellular models. It is important to note that while cellular models offer valuable insights, their simplicity lacks the intricate organ and tissue interactions crucial for a comprehensive physiological response. Therefore, conducting in vivo studies becomes pivotal for a more integrated understanding of how these extracts could potentially ameliorate diseases.

## 4. Materials and Methods

### 4.1. Chemicals

Unless otherwise indicated, the reagents employed were supplied by Sigma-Aldrich Corp. (Milan, Italy).

### 4.2. Plant Collection and Extraction

**Figure 4:**
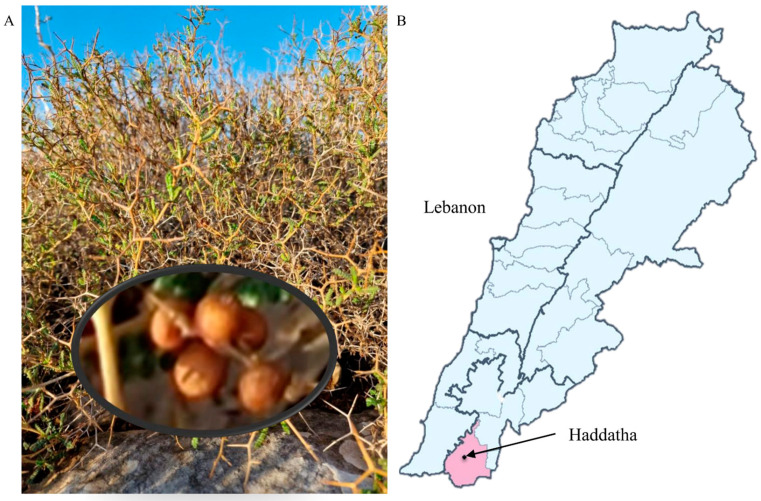
*S. spinosum* characteristics and distribution. *S. spinosum* plant in its natural environment: (**A**) the shrub and fruits; (**B**) the region of the fruit collection: Haddatha, South Lebanon.

*Sarcopoterium spinosum* (L.) Spach fruits (Figure 4) were collected in summer from the wild in South Lebanon, Haddatha (Latitude: 33°09′60.00′′ N, Longitude: 35°22′59.99′′ E). They were identified according to Prof. George Tohme, a taxonomist and president of Lebanese CNR. A voucher specimen (R5.36) was placed in the Lebanon National Herbarium at the Lebanese University Faculty of Sciences.

The fruits were dried for three weeks in a shaded, temperate environment. To prepare the ethanolic extract, a liter of 99% ethanol was mixed with 50 g of ground material. After 3 h, the mixture was filtered and then ethanol was evaporated, and the dry pellet was collected at 42 °C in a rotary evaporator (Heidolph Instruments, Schwabach, Germany). The MS characterization of the SEE characterized the polyphenolic profile of the extract (Table 1).

### 4.3. Protein Quantification

The protein content of the samples was quantified using a Bradford assay with bovine serum albumin (BSA) as a standard [42].

### 4.4. Cell Culture and Treatments

Human endothelial cells, the HECV cells (Cell Bank and Culture-GMPIST, Genoa, Italy), were cultured in Dulbecco’s modified Eagle’s medium High Glucose (D-MEM), supplemented with 2 mM Glutamine and 10% FBS at 37 °C in a humidified atmosphere with 5% CO_2_. For the experiments, cells were seeded in 35 mm^2^ plates, with approximately 40,000 cells per plate, and allowed to reach 70% confluency. We explored two experimental conditions:

- Counteraction Condition: The cells were first subjected to stimulation with H_2_O_2_ (30 µM for 24 h) to induce oxidative stress. Subsequently, they underwent post-treatment with either SEE, Cg, or Qu (10 µg/mL for 24 h);
- Protection Condition: The cells were pre-treated with either SEE, Cg, or Qu (10 µg/mL for 24 h) before being exposed to H_2_O_2_ (30 µM for 24 h);
- Non-treated normal cells (Ctrl) served as a crucial control to validate the efficacy of our experimental model. Furthermore, H_2_O_2_-insulted untreated cells (H_2_O_2_) were included as an inner control to compare and validate the outcomes. Each experiment was performed at least in quadruplicate.

### 4.5. Cell Viability Assessment

The anti-proliferative and/or cytotoxic activity of SEE and its main components was determined using the MTT assay. Cell viability was tested using the 3-(4,5-Dimethylthiazol-2-yl)-2,5-diphenyltetrazolium bromide (MTT) reduction assay [43]. Briefly, 2000 cells/well were seeded (200 μL volume) in a 96-well plate and incubated at 37 °C in humidified air with 5% CO_2_ for 24 h. After that, cells were treated with 10 µg/mL of either SEE, Cg, or Qu. After 24 h, 20 μL of MTT solution (5 mg/mL) was added to each well and incubated for 3 h. At the end, the medium was removed, the formazan was dissolved with 200 μL of isopropanol, and the absorbance was read at 570 nm.

### 4.6. ROS Production

Oxidant species production was quantified in situ using the oxidation of the cell-permeant 2′-7′-dichlorofluorescin diacetate (DCF-DA D-399, Thermofisher Scientific, Milan. Italy ) to 2′-7′-dichlorofluorescein (DCF) [44]. Briefly, cells were treated, collected, and then incubated with 1 μM DCF-DA in PBS prepared from stock 10 mM stock prepared in DMSO for 30 min at 37 °C in the dark. After that, cells were centrifuged, suspended in 2 mL PBS, and the fluorescence was read at 25 °C using a water-thermostated cuvette holder (λ_ex_ = 495 nm; λ_em_ = 525 nm) with a LS-50B fluorometer (Perkin Elmer, Waltham, MA, USA). Protein quantification was used for normalization. Results are expressed as percent fluorescence intensity relative to control. The results represent the average of at least three independent experiments in triplicate.

For fluorescence microscopy, the oxidant species were stained with 1 μM DCF-DA in PBS, and DNA with 2 µg/mL Hoechst 33342 (Thermofiher Scientific, Milan, Italy). Images were acquired at 20× magnification with optical and/or phase contrast microscopy using an inverted Olympus IX53 microscope (Olympus, Milano, Italy) equipped with a CCD UC30 camera and digital image acquisition software (cellSens standard 1.9). All images were processed using ImageJ 2.9.0/1.54c software.

### 4.7. Lipid Peroxidation

Thiobarbituric acid reactive substances (TBARS) assay [45] quantified the spectrophotometrically lipid peroxidation level, which is based on the reaction of malondialdehyde (MDA; 1,1,3,3-tetramethoxypropane) with thiobarbituric acid (TBA). Briefly, 250 µL of cell solution was incubated for 45 min at 95 °C with 500 µL of TBA solution (0.375% TBA, 15% trichloroacetic acid, 0.25 N HCl). Then, 750 µL of N-butanol was added and the organic phase was read at (λ_ex_ = 532 nm) in a UV-VIS spectrophotometer at 25 °C using Peltierthermostated cuvette holder.

### 4.8. Nitrite/Nitrate Levels

The nitric oxide level produced by HECV cells was determined using the Griess reagent following the previously described method [46]. The NO production uses spectrophotometric analysis of the end products, nitrites, and nitrates. Following treatments, nitrite accumulation was measured by incubating 500 mL media with 500 mL of griess reagent in the dark for 10 min, and the absorbance was recorded at 540 nm. The NaNO_2_ standard curve was used as a reference. Results were normalized with protein quantification and were expressed as (µmol NaNO_2_/mg sample protein).

### 4.9. GSH/GSSG Ratio

The ratio between the reduced (GSH) and oxidized (GSSG) glutathione is a frequently used indicator of oxidative stress in cells and tissues. In our cellular samples, the levels of GSH and GSSG were measured using the glutathione colorimetric assay kit (ZX-44100-96, Zelix, Berlin, Germany). Briefly, cells seeded in 100 mm^2^ plates were treated and then harvested in 1 mL of cold PBS, homogenized, and deproteinized with 5% 5-sulfo-salicylic acid. Total glutathione (GSH + GSSG) and GSSG levels were evaluated in the supernatant according to the manufacturer’s protocols. The absorbance of each sample was read in a UV-VIS spectrophotometer at 25 °C (λ_ex_ = 405 nm) using a plate reader, then the GSH level and the GSH/GSSG ratio were calculated as stated below:

GSH=Total glutathione−GSSG

$$\mathrm{GSH}/\mathrm{GSSG}=\frac{\mathrm{GSH}}{\mathrm{GSSG}}$$

### 4.10. Wound Healing Assay

For the wound healing assay [47], HECV cells were seeded on 35 × 10 mm^2^ tissue culture dishes and incubated until 100% confluence, then by using a p100 pipet tip, the cell monolayer was scraped, making two crossing straight lines to create a ‘‘scratch’’. Then, three views of the scratch were photographed using an inverted Olympus IX53 microscope (Olympus, Milan, Italy) and representative images were captured with a CCD UC30 camera and digital image acquisition software (cellSens standard 1.9). After scratching, the medium was replaced with fresh medium in the presence of each plant extract and the polyphenols or H_2_O_2_. The sets of images were acquired at 0 and 24 h. To determine the migration of HECV, the images were analyzed using ImageJ 2.9.0/1.54c software (http://imagej.nih.gov/ij/). The percentage of the closed area was measured by comparing t24 values with the value at t0. An increase in the percentage of closed areas indicated the migration of cells. Data are means ± S.D. of at least three independent experiments.

### 4.11. Protein Denaturation Assay

The protein denaturation assay was performed according to a classical method [48] with minor modifications. Briefly, either 2 mL (10 μg/mL) of the extract or of the NSAID diclofenac sodium (2-[(2,6-dichlorophenyl)amino] benzene acetic acid sodium salt) were mixed with 2.8 mL 1 M phosphate buffer (pH 6.4) and 0.2 mL 1% BSA. Then, they were incubated at 37 °C for 15 min. After that, the temperature was increased to 70 °C for 5 min. After cooling, the absorbance of the sample was read at 660 nm. The control (100% protein denaturized sample) was prepared as a 4.8 mL phosphate buffer with 0.2 mL 1% BSA. The percentage inhibition of BSA denaturation was calculated as stated below:

$$\%\;\mathrm{BSA}\;\mathrm{inhibition}=100*\left(\frac{\mathrm{Ab}\;\mathrm{control}-\mathrm{Ab}\;\mathrm{sample}}{\mathrm{Ab}\;\mathrm{control}}\right)$$

### 4.12. Statistical Analysis

The data presented in this study are expressed as means ± standard deviation (S.D.) and are the result of an analysis derived from a minimum of three independent experiments. To evaluate the significance of the observed differences, statistical analysis was performed by comparing the means of the different treatments using the ANOVA statistical method. Further detailed comparisons were conducted through Tukey’s post-test, which is particularly effective for identifying specific differences between multiple groups. The statistical analyses were carried out using GraphPad version 8.0.1 Software (GraphPad Software Inc., San Diego, CA, USA).

## 5. Conclusions

The present study is among the first and rare scientific investigations focused on the fruits of *S. spinosum* instead of the more investigated roots. An essential aspect of our approach is the emphasis on eco-sustainability. Indeed, by directing our interest on the fruits rather than on the roots, we could protect the plant from excessive uprooting, ensuring the preservation of its ecological integrity, recognizing the importance of maintaining the plant’s well-being and ensuring its long-term survival.

The other important conclusion is that *S. spinosum* fruits are rich in a panel of PPs, which are effective in playing antioxidant and anti-inflammatory roles in a simplified model of endothelial dysfunction. Moreover, our findings indicate that corilagin and quercetin can be the main players in sustaining the SEE’s beneficial effects.

Last but not least, our study explores various modalities of SEE administration, mirroring both habitual integration into daily routines and targeted use during periods of illness. Our findings demonstrate the efficiency of both modalities, highlighting the versatile and effective nature of SEE and its PPs in promoting health and mitigating disease risks. Consequently, we propose that this plant with its components could serve as a promising nutraceutical supplement for both preventing and treating metabolic and chronic diseases linked to oxidative stress.

## Figures and Tables

**Table 1:** Major polyphenols identified in the ethanolic extract from *S. spinosum* fruit using HPLC-MS/MS in the negative ionization mode and their respective abundance.

RT (min)	[M-H]^−^	MS/MS Fragments	Identified PPs	Classification	%
13.5	935	633/301/897	Casuarictin isomer	Ellagitannins	15.0
15.4	935	633/301/897/783	Casuarictin isomer	Ellagitannins	8.9
17.9	477.1	301	Quercetin glucuronide	Flavonoids	7.0
12.2	935	633/301	Castalagin/Vescalagin	Ellagitannins	6.7
7.5	783.2	633/301	Pedunculagin	Ellagitannins	4.8
5.6	633.1	301/463	Corilagin	Ellagitannins	3.3
22.6	709.3	501/663	23-hydroxytormentic acid ester glucoside [M+HCOO]^−^ isomer	Triterpenoids	2.2
22.8	707.3	499/661	Di-reduced 23-hydroxytormentic acid ester glucoside [M+HCOO]^−^	Triterpenoids	1.8
25.6	503.2	485/471/453/441	23-hydroxytormentic acid	Triterpenoids	1.2

## Data Availability

Data are contained within the article.
